# Development and pilot feasibility study of a health information technology tool to calculate mortality risk for patients with asymptomatic carotid stenosis: the Carotid Risk Assessment Tool (CARAT)

**DOI:** 10.1186/s12911-015-0141-y

**Published:** 2015-03-24

**Authors:** Adrienne E Faerber, Rebecca Horvath, Carey Stillman, Melissa L O’Connell, Amy L Hamilton, Karina A Newhall, Donald S Likosky, Philip P Goodney

**Affiliations:** The Dartmouth Institute of Health Policy and Clinical Practice, Lebanon, NH USA; Section of Vascular Surgery, Dartmouth-Hitchcock Medical Center, Lebanon, NH USA; The Center for Health Outcomes and Policy, University of Michigan, Ann Arbor, MI USA

**Keywords:** Carotid artery narrowing, Surgical decision-making, Health information technology, Risk calculator

## Abstract

**Background:**

Patients with no history of stroke but with stenosis of the carotid arteries can reduce the risk of future stroke with surgery or stenting. At present, a physicians’ ability to recommend optimal treatments based on an individual’s risk profile requires estimating the likelihood that a patient will have a poor peri-operative outcomes and the likelihood that the patient will survive long enough to gain benefit from the procedure. We describe the development of the CArotid Risk Assessment Tool (CARAT) into a 2-year mortality risk calculator within the electronic medical record, integrating the tool into the clinical workflow, training the clinical team to use the tool, and assessing the feasibility and acceptability of the tool in one clinic setting.

**Methods:**

We modified an existing clinical flowsheet with the local electronic medical record for the CARAT risk model. To understand how CARAT would fit into the existing clinical workflow, we observed the clinic and talked with the clinical staff to develop a process map for the existing clinical workflow. CARAT was completed by the clinic nurse for patients identified on the clinic schedule as having carotid narrowing. We analyzed post-implementation assessment in two ways: quantifying the proportion of eligible patients with whom CARAT was utilized, and surveying surgeons to understand the impact of CARAT on decision-making and clinical workflow.

**Results:**

With minimum investment of institutional resources, we were able to produce a workable tool and pilot the tool in our clinic within a 6 month time period. Over 4 months, 287 patients were seen in the clinic with carotid narrowing, and clinic staff completed CARAT for 195 (68%). Per-surgeon completion rates ranged from 29 to 81%. Most patients (191 of 195, 98%) patients had a low 2-year calculated mortality risk. Most surgeons believed the risk assessment aligned with their expectations of patient predicted risk.

**Conclusions:**

We successfully integrated CARAT into the existing electronic medical record and have preliminary evidence that CARAT can be a valuable tool for evaluating mortality risk for patients with diseased carotid arteries. Accuracy of the risk calculations must be evaluated in larger, multi-center studies.

## Background

For patients with stenosis (i.e. narrowing) of the carotid arteries, revascularization with surgery or stenting to relieve the blockage has an important benefit - reducing the risk of future stroke [1-4]. Evidence from randomized trials is clear for “symptomatic” patients who have already suffered a minor stroke due to these blockages. These patients have a greater than 20% annual risk of subsequent stroke without carotid revascularization, so nearly all patients choose revascularization to help limit their chances of suffering another, potentially more debilitating stroke [5-8].

However, revascularization is also recommended for patients with “asymptomatic” carotid stenosis – where patients have narrowed carotid arteries but have not had any symptoms such as a preceding stroke [6,8]. Patients with asymptomatic carotid stenosis have a much lower annual risk of stroke than those who have already had an event – approximately 3% per year in each year of the patient’s remaining life expectancy. The absolute benefit of revascularization is uncertain for many patients with asymptomatic carotid stenosis [6]. Determining the optimal treatment choice for a patient with asymptomatic carotid stenosis must carefully weigh several competing risks: the up-front risks of revascularization, the long-term risk of stroke, and the patient’s life expectancy [9-11]. While the short-term risks have been described, the long term risks are less well known. At present, a physicians’ ability to recommend optimal treatments based on an individual’s risk profile can be difficult, because they must quickly integrate two complex risks: the likelihood that a patient will have a poor peri-operative outcome, and the likelihood that the patient will survive long enough to garner benefit from the procedure [6-8].

Therefore, we developed and pilot-tested a health information technology tool to help physicians and patients better understand these two risks [12]. First, we used short-term surgical risk stratification, from the detailed patient and procedural variables present in our national vascular registry, the Vascular Quality Initiative (VQI) to help clinicians identify those patients who are likely to fare poorly with carotid revascularization. Second, we used five-year longitudinal follow-up from a linked carotid-Medicare claims VQI dataset to assess the effectiveness of carotid revascularization in preventing stroke during the patient’s remaining life expectancy. In a previous publication, we described the variable construction and preliminary validation of the carotid risk assessment tool (CARAT) [Jessica B. Wallaert, Karina A. Newhall, Bjoern D. Suckow, Benjamin S. Brooke, Min Zhang, Adrienne E Faerber, Donald Likosky, Phillip P Goodney: Development of a model to predict long-term survival and stroke-related costs following asymptomatic carotid revascularization, under review]. In this manuscript, we describe the development of CARAT into a clinician decision support tool within the electronic medical record (EMR), integrating the tool into the clinical workflow, training the clinic staff to use the tool, and assessing the feasibility and acceptability of the tool in one clinic setting. We hypothesized that it would be feasible to implement CARAT within the EMR and clinical workflow, that the clinic staff would use the tool, and that the tool would be acceptable to surgeons.

## Methods

### The CARAT risk model

In previous work, we describe the generation and validation of the risk model upon which CARAT is based [Jessica B. Wallaert, Karina A. Newhall, Bjoern D. Suckow, Benjamin S. Brooke, Min Zhang, Adrienne E Faerber, Donald Likosky, Phillip P Goodney: Development of a model to predict long-term survival and stroke-related costs following asymptomatic carotid revascularization, under review]. CARAT uses individual characteristics of the patient with a carotid artery condition (age, smoking status), comorbidities (diabetes, CHF, COPD, renal function), statin use, and extent of carotid narrowing to calculate a risk score that predicts 2-year mortality (Table 1). The risk score ranges from 0 to 20, where lower scores indicate lower 2-year mortality. Scores are stratified into three validated risk tiers: 0–10 is “safe for surgery”, 11–14 is “consider surgery carefully”, and 15+ is “surgery may not be indicated based on limited life expectancy,” (Table 2).

**Table 1:** **Factors associated with increased 2-year mortality following carotid revascularization and the points each factor is given in calculating the CARAT risk score**

**Patient characteristic**	**Points**
1. Age (in years)	
<70	0
70-79	2
≥80	4
2. Insulin-requiring diabetes	2
3. CHF; Asymptomatic	2
CHF; Symptomatic	4
4. COPD; Mild/moderate	2
COPD; Oxygen dependent	4
5. Smoker; Past history or current	1
6. Renal function; eGFR < 60	3
Dialysis dependent	4
7. Contralateral ICA stenosis	
<50%	0
50-79%	1
80-99%	1.5
Occluded	3
8. Not on statin	1

**Table 2:** **Risk scores, mortality prediction and decision support messages for CARAT**

**Risk score**	**Predicted 2-year mortality**	**Decision support message**
0 to 2	2%	Safe for Surgery
3-4	3%	
5-6	5%	
7-8	8%	
9-10	13%	
11-12	21%	Consider Surgery Carefully
13-14	34%	
15-16	51%	Surgery May Not Be Indicated, Based on Limited Life Expectancy
17-18	70%	
19-20	88%	
>20	99%	

Risk scores are calculated from the factors in Table 1.

### Developing CARAT in an electronic medical record

Our previous experience with designing risk assessment tools showed us that direct EMR integration (versus a pen-and-paper form) would offer better usability and utility for CARAT, hence focused on developing CARAT for an existing EMR. We chose the EMR currently used in our clinical setting, which is Epic. We worked in collaboration with Epic designers to modify an existing clinical flowsheet for the CARAT risk model, which provided a simpler and quicker process for implementing the tool in the EMR.

### Engaging key stakeholders

Several key stakeholders were engaged in developing CARAT within our EMR. These stakeholders varied from EMR designers and analysts who modified the existing flowsheet for our risk tool, clinic nurses who collected and entered the necessary data elements, and surgeons who interpreted the output from CARAT. Each stakeholder was offered key opportunities to participate in the process development and provide feedback and input. This process occurred over four months via group meetings, surveys, and individual consults. Meetings with EMR developers allowed us to integrate our findings into the electronic record in a user-friendly fashion. Discussions with clinic staff (surgeons, nurse practitioners, nurses, and medical assistants) reviewed the steps necessary to use the tool. Finally, discussions with patient development partners reviewed the steps necessary to use the tool during a clinical encounter.

### Workflow integration & clinic staff training

**Figure 1:**
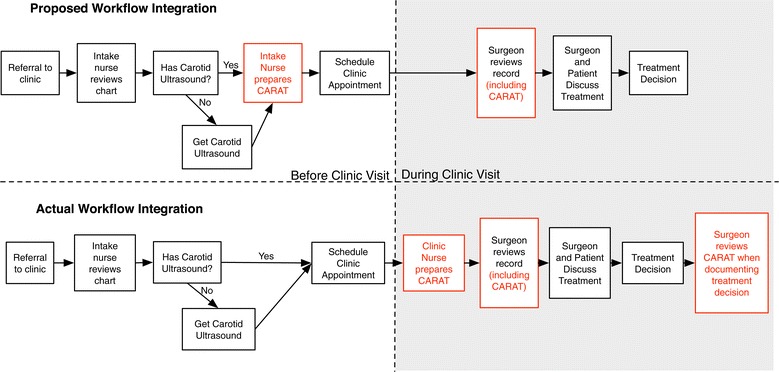
**Proposed and actual use of CARAT in clinic workflow.** As proposed, CARAT would be used early in the workflow and completed by an intake nurse and reviewed by the surgeon before the clinical encounter. In practice, CARAT was completed by the clinic nurse and reviewed by the surgeon before or after the consultation.

The pilot feasibility study of CARAT as a medical calculator took place in a tertiary vascular surgical clinic at an academic medical center in New Hampshire, United States. To understand how CARAT would fit into the existing clinical workflow, we observed the clinic and talked with the clinic staff to develop a process map for the existing clinical workflow (Table 3). From the process maps, we discussed possible ways to integrate CARAT into the clinical workflow. In the workflow (Figure 1), CARAT was completed by the clinic nurse for patients identified on the clinic schedule as having a carotid condition (indicated by a completed carotid ultrasound). The resulting risk score was then available as part of the medical record (along with the ultrasound, medical history, and other relevant laboratory tests) for the surgeon to review before discussing treatment options with the patient. A surgeon or nurse practitioner provided the risk estimate to the patient. Discussions regarding the language used to communicate risks to the patients were particularly challenging to develop, as surgeons expressed concerns regarding liability and decision-making. For example, surgeons did not want the language to limit their ability to offer surgery to patients who were potentially high-risk, but had clinical indications that pre-empted the advice offered by our risk prediction tool.

**Table 3:** **Clinic staff**

**Clinic staff**	**Number working in clinic**
Vascular surgeons	9
Nurse practitioners	1
Nurses	3
Medical assistants	2
Scheduling assistants	2
Receptionists	1

The clinical staff was trained to use CARAT in a group training session followed by individual consults with the project team as implementation rolled out. Training sessions included clinic nurses, medical assistants, and receptionists. Training session objectives were to illustrate how to complete CARAT in the EMR, provide opportunities for the clinical team to complete CARAT in a “sandbox” environment, and solicit feedback from the clinical team on integrating CARAT into the workflow.

### Feasibility and acceptability of CARAT

Once CARAT had been created and integrated into the EMR within our vascular surgery clinic, we evaluated the feasibility and acceptability of the tool. We analyzed post-implementation assessment in two ways: quantifying the proportion of eligible patients with whom CARAT was utilized, and surveying surgeons to understand the impact of CARAT on decision-making and clinical workflow. This survey asked surgeons about using CARAT, whether CARAT results were in concordance with their expectations, when CARAT would fit in the workflow, and the value CARAT could add to clinical encounters. These data were evaluated using existing clinical information systems within our EMR, as well as surveys distributed to the surgeons who saw patients within our clinic (n = 10). All analyses were performed using SAS (Cary, NC) and STATA 12 MP (College Station, TX). Dartmouth’s Committee for the Protection of Human Subjects approved our research protocol and approved the waiver of need for informed consent.

## Results

### Developing CARAT in an electronic medical record

At our institution, the development process for most new tools in the EMR involves a lengthy process of specifying inputs, designing and testing the user interface, integrating existing EMR data with the new tool and designing outputs like reports or analysis datasets. Though this process would produce a tool with good usability and integration, we opted instead to take a faster approach to development by modifying an existing function within the EMR that would suit our needs. This meant that we did not have an ideal interface, nor did we have customized reports on the use of the tool. But, with minimum investment of institutional resources, we were able to produce a workable tool and pilot the tool in our clinic within a 6 month time period.

### Workflow integration & clinical team training

It was anticipated that the CARAT score would be calculated during the referral and intake process, before the clinic visit to discuss treatment options for carotid stenosis. This approach would potentially allow surgeons to know the patient’s 2-year estimated post-surgical mortality before scheduling the patient for an appointment. However, after reviewing existing clinic workflow, the clinic staff chose to integrate CARAT within the nursing intake of the patient on the day of the clinic visit. Instead of being completed by a nurse at the time of referral, CARAT was completed by a nurse on the day of the visit as part of the standard nursing assessment and collection of data prior to patient being seen by the surgeon. Additionally, we had intended CARAT to be included in the medical record before the day of the visit to allow surgeons time to review CARAT before the clinical encounter and to tailor their discussion of treatment options based on the results of CARAT. With CARAT integrated as a component of usual vitals of the carotid patient upon entry to clinic, the data was available to the surgeon if s/he chose to view prior to or during time with the patient in the exam room. There was not observed consistency in patterns of behavior amongst surgeons and nurse practitioners in viewing the results of CARAT: some surgeons and nurse practitioner viewed the results before consultation, allowing them to tailor their discussion of treatment options, while others used the consultation as an opportunity to gather more information and consulted with the completed CARAT after the consultation. A few surgeons were able to tailor their discussion of treatment options, although few described this as a discrete disadvantage during our post-implementation survey.

### Feasibility and acceptability of CARAT

We pilot tested the CARAT tool by examining how commonly it was used by providers, and surveying providers regarding the utility of the tool. For four months after the clinic began using the tool, we assessed the feasibility of our CARAT tool by quantifying how often the tool was used. We performed a clinical audit to capture the number of patients eligible for CARAT assessment as well as the number of patients in whom CARAT was actually performed and viewed by the physician.

Over the four month assessment period, 287 patients were seen in the clinic who had a preliminary diagnosis of carotid stenosis which met the threshold for surgery (greater than 70% blockage), and had a recent ultrasound confirming the degree of stenosis, so were potentially eligible for CARAT. Clinic staff completed the CARAT for 195 (68%). All of the surgeons and nurse practitioners in the clinic (n = 10) used CARAT for their patients, and completion rates ranged from 29 to 81%. Completion rates were most variable for surgeons that saw fewer than 10 patients during the study period (range 29% to 80%). For surgeons and nurse practitioners that saw more than 10 patients, completion rates were between 42 to 81%. Almost all patients were assessed as having low risk (scores from 1–10 points) on CARAT. In our population of elective patients referred for carotid artery stenosis, only 4 had medium risk (11–14), and none had high risk characteristics during our study period. The distribution of patients among the three risk groups was similar to the distribution of scores in model development [Jessica B Wallaert KAN, Bjoern D. Suckow, Benjamin S. Brooke, Min Zhang, Adrienne E Faerber, Donald Likosky, Phillip P Goodney: Development of a model to predict long-term survival and stroke-related costs following asymptomatic carotid revascularization, under review].

Finally, we sought to assess the acceptability of CARAT by surveying the surgeons and nurse practitioners who saw patients in the clinic where CARAT was implemented. Eight of the 10 surgeons and nurse practitioners who used the CARAT tool completed a survey. Three surgeons or nurse practitioners did not recall entering data into the CARAT tool itself, because other members of the clinic staff (nurses, medical assistants) completed the tool for them. Of the remaining 5 surgeons or nurse practitioners who recall using CARAT, 4 of 5 believed that CARAT results aligned with their expectations, while one was unsure if the risk prediction generated by CARAT aligned with their expectations.

We asked surgeons and nurse practitioners questions to determine their views about the optimal time during the episode of care for carotid artery stenosis to use CARAT. Options for use of the tool varied from the time of primary care provider visit through the occurrence of specialty services such as consultation, imaging, or even just before surgery. Nearly all surgeons or nurse practitioners agreed that primary care was not the appropriate place (7 of 8) because questions regarding the appropriateness of surgery could not be reliably answered in that setting. However, surgeons and nurse practitionersdisagreed as to the optimal time period to use the CARAT tool: upon referral to vascular services (3 of 8 supporting), at time of testing (3 of 8), during the pre-visit chart review (2 of 8), or when discussing treatment options (2 of 8).

Lastly, we asked about the value that CARAT can add to assessing risk of the patient with asymptomatic carotid stenosis. All surgeons and nurse practitioners (8 of 8) agreed that CARAT provides value in adding objective evidence to clinician recommendations. Further, 7 of 8 stated that CARAT supports informed decision-making by the patient, 4 of 8 stated that CARAT increased their confidence in making a recommendation in favor of - or against - surgery. Half of surgeons and nurse practitioners (4 of 8) felt that CARAT provided value in building registry data for advancing outcomes-based research. And finally, when we asked surgeons and nurse practitioners to describe positive attributes of CARAT being embedded in the patient record, 6 of 8 reported the ability to document of the values generated by CARAT, 5 of 8 reported the ability to share these data among medical providers, and 4 of 8 said ease of access. No negative comments about EMR integration were offered by the surgeons and nurse practitioners.

## Discussion

In this project, we developed the Carotid Risk Assessment Tool (CARAT), a risk calculator, into our electronic medical record (EMR), integrated the tool into clinical workflow, trained staff to use the tool and evaluated feasibility and acceptability of CARAT. CARAT is designed to help physicians make better choices when faced with decisions surrounding revascularization for asymptomatic carotid artery stenosis.

The first two steps -development and EMR integration necessitated a multidisciplinary approach, including statistical consultants, EMR technology development experts, implementation scientists, clinical faculty, and patients. While our third step – implementation of the tool in a clinical setting – was also multidisciplinary, these team members were the more common members involved in healthcare related interactions, such as surgeons, nurses, and clinic staff. We found that this team - which incorporated team members from heterogeneous backgrounds working together in a multi-faceted approach – was able to effectively and efficiently design and implement our Health IT tool aimed at limiting unnecessary carotid revascularization.

Post-implementation discussions among our team members led us to agree that our efforts to design and implement CARAT were successful because we used two key approaches: team-based interactions and workflow integration. First, our team-based interaction meant that we worked directly with our Health IT implementation team to generate an interface where the CARAT data could be easily and efficiently entered in a manner that did not interfere with clinic flow. Second, in terms of workflow integration, we allocated time and dedicated effort within the CARAT implementation process for nursing flow and education in garnering the necessary information to generate the risk score in CARAT (Figure 1). This allowed the “work” of CARAT data collection to be incorporated into clinic workflow as a distinct, new element, with a specific team member assigned to this task, yet simply assimilated into existing activities. This helped us have accountability in this effort across a multidisciplinary team [13]. Further, we designed the CARAT EMR interface in Epic to allow the physicians and other health care providers to quickly and promptly gain access to the necessary information contained in CARAT. This allowed all team members – not just the surgeons using the tool – to know which elements were necessary to collect, and how they were integrated into the tool. The combination of these three approaches allowed us to develop and implement CARAT in a fashion that quickly and conveniently conveyed the necessary information to providers.

### Next steps: examining the effect of EMR-based decision tools on patient decisions

While it is challenging to design EMR-based decision-tools in a way so that providers actually use the tools, it is equally important to design the tools so that key stakeholders – project leaders, collaborators, and project supporters – can determine if the tools are being used, and used successfully. Our work was able to easily accomplish the former, as it was clear which patients had a clinical encounter involving carotid stenosis, and we could easily audit Epic for CARAT scores. The latter – determine if the tool was used successfully, and actually affected clinical decisions – remains undescribed at present. Additionally, this pilot study did not evaluate the extra time or cost needed to use CARAT, leaving unanswered whether or not this intervention is cost-effective. In other words -- did the use of CARAT result in fewer unnecessary carotid interventions? Did patients and providers feel that they had better quality of shared decision making? Was decisional regret less common in patients who used the decision tool? How much extra cost was incurred using CARAT? Our current study did not answer these questions. However, we have taken the initial steps in building the foundation necessary for these experimental approaches.

Our future work aims to explore these questions using validated instruments and approaches, as well as tools that will educate not only providers, but integrate with patients directly (optiongrids.org). We will translate the lessons learned in building the initial CARAT tool – in terms of the tool itself, how it was implemented, and how it was studied – in our next patient-centered version of CARAT.

### Limitations

Our study has several limitations. First, we implemented CARAT in a single center, which used a single EMR (Epic). While this implementation was successful in allowing providers to use CARAT, broader implementation in multiple centers will be necessary to ensure our approach is generalizable. Second, in implementing CARAT in this clinic, we needed to be prescriptive as to where the tool would fit into the clinic workflow, and this may limit the ability of the clinic staff to use the tool in the way they find most useful. Future studies will allow more flexibility in use of the tool to fit into existing workflows and to be used when perceived as useful. Third, we only have information about patients for whom CARAT was completed, and no information about the patients for whom CARAT was not completed, thus affecting the generalizability of the patient-level findings. Future studies incorporating a control arm will address this limitation. Fourth, the survey was fielded to a small number of providers and the results were heterogeneous. Because of the small size of the clinic staff, we will need to repeat the survey in a larger multicenter trial to obtain generalizable results about different members of the clinic staff. Fourth, as we outlined above, the effect of the CARAT tool on utilization and decision quality remains to be performed, and will benefit from the multisite settings we will develop in our future work. We will focus these questions not only on the provider’s perception of utility, but also incorporate patient perceptions of the utility of the tool as well. And finally, while our preliminary analyses suggested that providers understood the language and presentation in the risk tool, we will need definitive examination of how patients and their providers understand and interpret the information the CARAT tool provided during the clinical interaction.

## Conclusions

In this project, we developed the Carotid Risk Assessment Tool (CARAT), a risk calculator, into our electronic medical record (EMR), integrated the tool into clinical workflow, trained staff to use the tool and evaluated feasibility and acceptability of CARAT. A multidisciplinary approach was an important aspect of implementing CARAT within our center, and helped facilitate effective adoption of the tool during clinical encounters with patients with asymptomatic carotid artery stenosis. Our future work will expand the scope and the depth of evaluation of our tool, as we strive towards our goal of improving decision quality for patients faced with difficult surgical challenges.
